# Supplementary material for: Can strengthening digital infrastructure enhance productivity in the cultural industry? evidence from Tibet
Source: PLoS One. 2025 Jan 24;20(1):e0317366. doi: 10.1371/journal.pone.0317366 (PMC11759985; doi:10.1371/journal.pone.0317366)
Supplement: Attachment — Submitted filename: Response to Editor and Reviewer_20241203.pdf [file pone.0317366.s004.pdf]

December 3, 2024
Response to Editor and Reviewer

Dear Reviewers,

We sincerely thank you for your thoughtful and constructive feedback. Your insights have been invaluable in enhancing the academic rigor and conceptual clarity of our manuscript. Below, we address the specific comment and outline the corresponding revisions.

Reviewer #1

Comment: Total Factor Productivity (TFP) is not a theoretical framework but rather a concept used in economics to measure the efficiency of all inputs in the production process. TFP is based on theoretical foundations, such as growth theory.

Response: We greatly appreciate this crucial observation and have thoroughly revised the manuscript to ensure TFP is accurately positioned as an economic measurement tool. The key revisions are as follows:

1. Clarification of TFP's conceptual role:

We have refined the description of TFP throughout the manuscript, emphasizing that it serves as a tool to quantify the contributions of resource optimization and technological progress to production efficiency. While TFP itself is not a theory, we highlight its alignment with the theoretical frameworks of growth theory and innovation diffusion theory, which provide the logical foundation for its application in this study.

2. Distinction between theory and methodology:

We carefully reviewed and revised the manuscript to distinguish theoretical frameworks from methodological tools. For instance, the theoretical foundation section now focuses on the roles of growth theory and innovation diffusion theory in guiding the analysis, while TFP is presented as a practical tool to evaluate production efficiency. Specific examples of its application in resource allocation, technological progress, and regional development are detailed in the methodology and results sections.

3. Specific revisions to avoid confusion:

- In the abstract and introduction, we removed any phrasing that might misrepresent TFP as a theoretical framework, emphasizing its role as a measurement tool and clarifying its connection to the theoretical foundations.
- In the theoretical foundation section, we expanded the discussion of growth theory and innovation diffusion theory to underscore their roles in supporting the research.
- Throughout the manuscript, we enhanced the explanation of TFP's specific application in assessing production efficiency, ensuring the logical relationship between theory and methodology is explicit and rigorous.

These revisions aim to present TFP's academic positioning with greater accuracy and ensure its integration with the theoretical framework is both logical and precise.
We sincerely appreciate the reviewer's insightful comment, which has significantly contributed to the improvement of our manuscript. These revisions have clarified the

conceptual framework and strengthened the manuscript’s theoretical and methodological alignment. We hope that the revised manuscript meets the journal’s high standards, and we remain open to any further suggestions or feedback.

Thank you again for your valuable input！

Sincerely,

Yuanyuan Li, Qianqian Du, Guihua Ma , and Gazang Caidan
School of Economics and Management, Tibet University
Email: 291253725@qq.com
