# Supplementary material for: Can strengthening digital infrastructure enhance productivity in the cultural industry? evidence from Tibet
Source: PLoS One. 2025 Jan 24;20(1):e0317366. doi: 10.1371/journal.pone.0317366 (PMC11759985; doi:10.1371/journal.pone.0317366)
Supplement: Attachment — Submitted filename: PONE-D-24-29366_comments.docx [file pone.0317366.s002.docx]

This paper developed an empirical study to analyze how digital infrastructures can increase the productivity of cultural industries by adjusting industrial agglomeration, improving the efficiency of goods circulation and narrowing the consumption gap between urban and rural areas in Tibet, China. In general, this research fits in the main theme of the journal, but still requires several revisions. Below are some specific comments:

1. New quality productivity is a concept put forward by China's high-quality development, not a theory, so the author should correctly understand the expression.
2. The contribution of the article needs to be further highlighted.
3. The “Theoretical Mechanisms and Research Hypotheses” section needs to be strengthened with references to support the hypotheses.
4. The tables in the article are vague and are not placed in the appropriate place in the article.
5. Why does the sentence“Here is the English translation for your description of the use of statistical software and regression analysis:” appear in the article? Please critically check the content of the article expression
6. In table 8, what is the significance of A? Adding a note or applying the full name would be more beneficial to the reader's understanding
7. In table 9, the significance of * is not clearly indicated, does it mean a statistically significant level as in the previous table or is it a confidence level as described in your article?
8. In the Robust test, firstly, the authors substitute the dependent variable by replacing it, but are the two variables substitutable for each other? Is there any evidence for this? Secondly, the author applies IV to eliminate endogeneity, but does not verify the validity of IV, is the IV you chose appropriate?
9. The description of the results section of the article is not clear and concise enough, there are too many tables, and the formatting of the tables should be improved to make them clearer and more intuitive.
10. The article lacks further discussion of the main results, which need to be analyzed in the ‘Discussion’ section, considering literature comparisons.
11. The academic writing of this manuscript needs to be improved. There are a few problems including format, grammar, logic flow, specific writing style, etc. please check and revise accordingly.
